# The Transition From Sunitinib Monotherapy to Modern First-Line Tyrosine Kinase Inhibitor (TKI)-Containing Regimens in Metastatic Clear-Cell Renal Cell Carcinoma

**DOI:** 10.7759/cureus.115539

**Published:** 2026-08-31

**Authors:** Sravani Manda, Shruti Singh

**Affiliations:** 1 Internal Medicine, Saba University School of Medicine, The Bottom, BES; 2 Biochemistry and Genetics, Saba University School of Medicine, The Bottom, BES

**Keywords:** first-line therapy, immune checkpoint inhibitors, progression-free survival, renal cell carcinoma, sunitinib, tyrosine kinase inhibitors

## Abstract

Sunitinib historically served as a standard first-line treatment for metastatic clear-cell renal cell carcinoma (RCC), but its contemporary use is limited by inferior efficacy compared with several modern combination regimens, together with cumulative toxicity and acquired resistance. Newer tyrosine kinase inhibitor (TKI)-based treatments have emerged as alternatives, demonstrating improved efficacy but also substantial and regimen-specific toxicity burdens. Contemporary treatment recommendations now favor modern first-line regimens over sunitinib for most patients with advanced or metastatic clear-cell RCC. This review evaluates the clinical evidence supporting the transition from sunitinib monotherapy to contemporary first-line TKI-containing regimens.

PubMed and Embase were searched for clinical trials evaluating sunitinib compared with contemporary first-line TKI-based or TKI-containing regimens in previously untreated advanced or metastatic clear-cell RCC. Twenty-two records were identified through database searching, and one additional publication was identified during peer review. After removal of five duplicates, 18 records were screened. Ten publications representing six unique clinical trials met the eligibility criteria and were included in the qualitative synthesis. Extracted data included study design, patient population, treatment arms, progression-free survival (PFS), overall survival (OS), objective response rate (ORR), and adverse events (AEs).

Across the included publications, TKI-containing first-line regimens consistently improved PFS compared with sunitinib. Cabozantinib improved median PFS compared with sunitinib in intermediate- or poor-risk advanced clear-cell RCC [8.6 vs. 5.3 months; hazard ratio (HR) 0.48]. Nivolumab plus cabozantinib improved median PFS [16.6 vs. 8.3 months; HR 0.51] and ORR (55.7% vs. 27.1%). Pembrolizumab plus axitinib improved 12-month OS (89.9% vs. 78.3%; HR 0.53), median PFS (15.1 vs. 11.1 months; HR 0.69), and ORR (59.3% vs. 35.7%). Lenvatinib plus pembrolizumab demonstrated durable benefit in CLEAR, with median PFS of 23.9 versus 9.2 months (HR 0.47), ORR of 71.3% versus 36.7%, and a final OS HR of 0.79. Benmelstobart plus anlotinib also improved median PFS (19.0 vs. 9.8 months; HR 0.53) and ORR (72% vs. 25%). OS benefit varied across trials, while high-grade adverse events were common, with safety definitions differing across studies.

The included evidence supports the transition away from sunitinib as the preferred first-line treatment for most patients with advanced or metastatic clear-cell RCC. However, sunitinib remains an evidence-based option for selected patients, including those with contraindications to immune-checkpoint inhibition, some patients with favorable-risk disease, and those with limited access to combination therapy.

## Introduction and background

Renal cell carcinoma (RCC) accounts for more than 90% of kidney malignancies and remains one of the most clinically significant urologic cancers due to its frequent progression to advanced or metastatic disease [1]. Although treatment options for localized RCC are primarily surgical, management of metastatic clear-cell RCC relies heavily on systemic therapy [1]. Historically, vascular endothelial growth factor (VEGF)-targeted tyrosine kinase inhibitors (TKIs), particularly sunitinib, were widely used as the standard first-line treatment for metastatic clear-cell RCC [1,2]. However, advances in molecular oncology and immunotherapy have expanded therapeutic options beyond sunitinib, resulting in improved disease control and survival outcomes in patients with metastatic clear-cell RCC [2,3].

RCC comprises several histologic subtypes with important therapeutic and prognostic implications. Clear-cell RCC is the most common subtype and accounts for approximately 65-70% of all RCC cases and forms the basis for most clinical trials evaluating systemic therapies [4]. Non-clear cell subtypes, particularly papillary RCC, remain comparatively understudied, though emerging evidence suggests treatment responses may differ across histologies [1].

Clear-cell RCC is strongly associated with loss of the von Hippel-Lindau (VHL) tumor-suppressor gene, which increases hypoxia-inducible factor (HIF) activity and promotes VEGF-driven blood-vessel formation (angiogenesis) [1,5]. Other pathways, including PI3K/AKT/mTOR and mesenchymal-epithelial transition factor (MET), also contribute to tumor progression and treatment resistance [6]. These mechanisms support the use of VEGF-targeted TKIs and, more recently, TKI-containing regimens that combine a TKI with an immune checkpoint inhibitor rather than relying on TKI monotherapy alone.

Management of RCC depends on tumor stage, histology, and patient-specific factors. While partial or radical nephrectomy remains the standard treatment for localized disease, systemic therapy is central to the management of advanced and metastatic clear-cell RCC [1]. Historically, sunitinib served as the standard first-line therapy for metastatic clear-cell RCC because of its ability to delay disease progression compared with earlier systemic treatments [2]. However, multiple major randomized clinical trials have since demonstrated improved outcomes with newer TKI-based and TKI-containing regimens compared with sunitinib.

These regimens have shown improvements in progression-free survival, overall survival, and response rates, leading to major changes in treatment recommendations. As a result, contemporary National Comprehensive Cancer Network (NCCN) guidelines now favor several modern first-line regimens over sunitinib for most patients with advanced or metastatic clear-cell RCC, with treatment decisions influenced by histology, patient-specific factors, and International Metastatic Renal Cell Carcinoma Database Consortium (IMDC) risk stratification, which categorizes patients into favorable-, intermediate-, or poor-risk prognostic groups [7].

As treatment recommendations have evolved, reviewing this therapeutic transition is important for understanding the changing role of sunitinib in modern clear-cell RCC management. The decline in sunitinib use reflects both the availability of alternative TKI therapy in selected populations and, more prominently, the emergence of TKI-containing combination regimens with improved outcomes compared with sunitinib monotherapy. Therefore, the objective of this review is to evaluate the clinical evidence supporting the transition from sunitinib monotherapy to contemporary first-line TKI-containing regimens for advanced or metastatic clear-cell RCC.

## Review

Methods

Search Strategy

A literature search was conducted in PubMed and Embase to identify primary clinical studies evaluating sunitinib compared with newer first-line tyrosine kinase inhibitor (TKI)-based or TKI-containing therapies in advanced or metastatic clear-cell renal cell carcinoma (RCC). The PubMed search strategy was: “Carcinoma, Renal Cell”[MeSH] AND “Sunitinib”[MeSH] AND “Progression-Free Survival”[MeSH] AND “Untreated”[All Fields]. Clinical trial and randomized controlled trial filters were applied. The final PubMed search was conducted on June 3, 2026. A supplementary Embase search was conducted on August 16, 2026, using “renal cell carcinoma” AND “sunitinib” AND “progression-free survival” AND “untreated,” with limits applied for full-text randomized controlled trials and Embase-indexed records. Studies were limited to English-language publications. No publication-date restriction was applied.

Study Screening and Eligibility

Studies were included if they were published in English, involved adult human subjects with advanced or metastatic clear-cell renal cell carcinoma, evaluated a TKI-containing regimen either alone or in combination with a monoclonal antibody in previously untreated patients, included sunitinib as a comparator treatment arm, and were designed as randomized controlled trials or clinical trials. Studies were excluded if they were systematic reviews, meta-analyses, editorials, commentaries, conference abstracts without full data, case reports, animal studies, preclinical or phase 0 trials, or studies not evaluating first-line systemic therapy in advanced or metastatic clear-cell RCC. These studies did not include sunitinib as a comparator, studies evaluating antibody-only experimental regimens without a TKI component, or studies involving previously treated advanced or metastatic disease. A single reviewer performed title and abstract screening and full-text eligibility assessment. A review protocol was not prospectively registered. One additional eligible publication identified during the peer-review process was assessed using the same eligibility criteria and incorporated into the qualitative synthesis.

Full-text articles meeting the eligibility criteria were included in the qualitative synthesis. Study characteristics, methodological limitations, and generalizability were qualitatively considered during evidence synthesis. The study selection process is summarized in the flow diagram (Figure 1) shown below:

**Figure 1:**
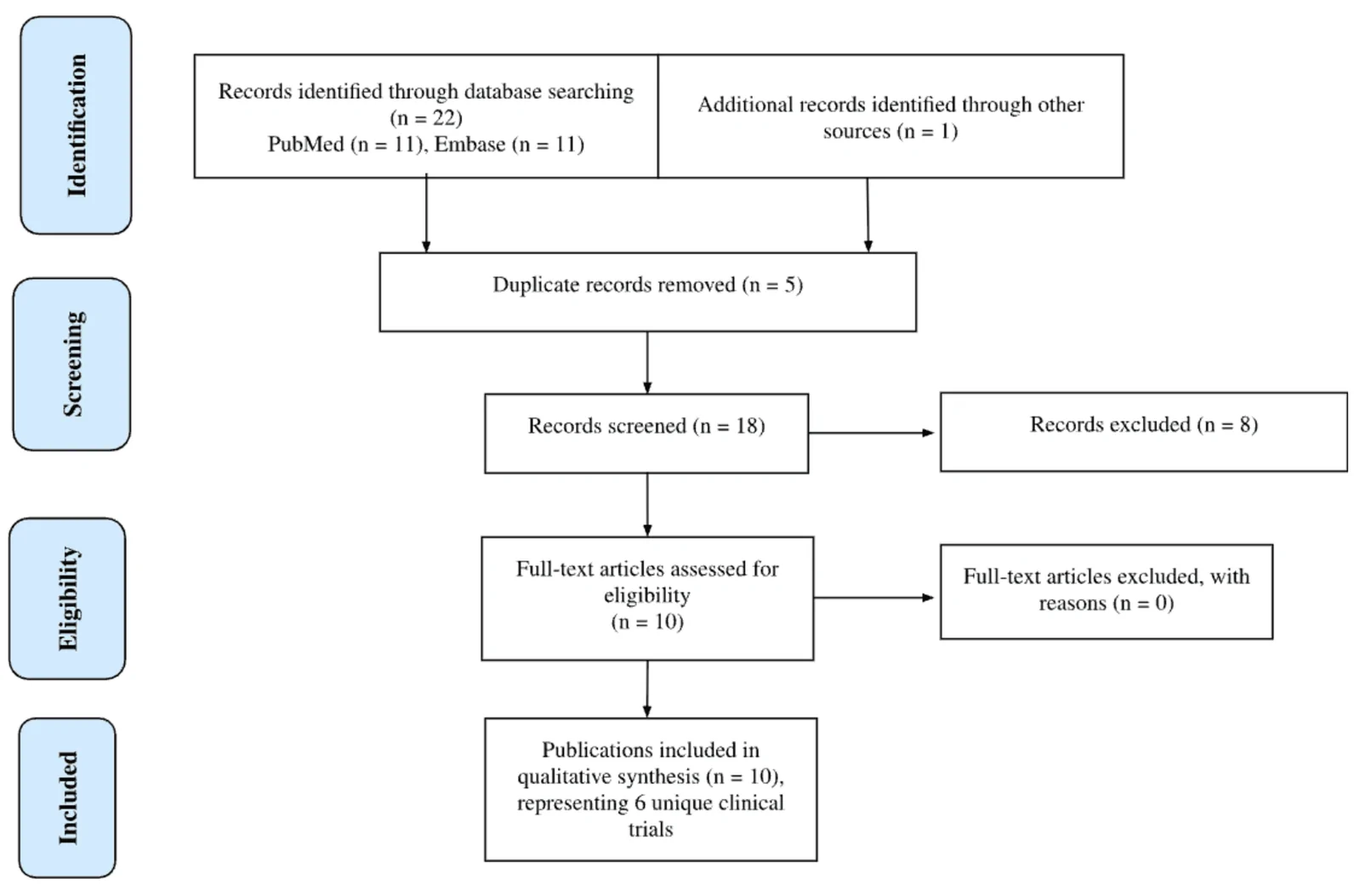
Flow diagram illustrating the study selection process

Data Extraction

Data extraction was performed by a single reviewer and was not independently duplicated by a second reviewer. Data from eligible studies were extracted and compiled into evidence tables (Appendices 1, 2, 3). When multiple publications reported data from the same underlying clinical trial, they were retained when they provided distinct primary, updated, or subgroup analyses; however, they were linked to the same trial and were not treated as independent patient populations. Extracted variables included citation details, author and publication year, study design, level of evidence, sample size, study population, key inclusion criteria, RCC histology, prior treatment status, risk group or performance status when reported, treatment interventions, follow-up duration, key outcomes, numerical findings, progression-free survival, overall survival, objective response rate, hazard ratios, confidence intervals, adverse-event definitions and rates, treatment discontinuation due to adverse events, treatment-related deaths, dose modifications or interruptions, immune-related adverse events when reported, and generalizability notes related to trial population, risk stratification, and subgroup limitations.

The evidence tables were then used to compare outcomes across publications and identify consistencies and differences in efficacy, safety, trial population, risk stratification, and generalizability related to sunitinib and TKI-containing regimens in clear-cell RCC.

Synthesis Approach

Because the included studies evaluated clinically distinct treatment strategies, findings were synthesized qualitatively rather than pooled. Evidence from TKI monotherapy comparisons was considered separately from TKI-containing combination regimens compared with sunitinib monotherapy. Interpretation emphasized within-trial treatment effects, and direct numerical ranking across separate trials was avoided because of differences in patient populations, follow-up duration, and outcome assessment.

Risk-of-Bias Assessment

Risk of bias was assessed using the Cochrane Risk of Bias 2 (RoB 2) tool for randomized trials. Because RoB 2 evaluates bias in a specific result rather than assigning a general quality rating to an entire study, the assessment focused on progression-free survival (PFS), a principal efficacy outcome reported across all six randomized trial programs included in this review. The effect of interest was the effect of assignment to intervention. Multiple publications arising from the same underlying trial were considered together and were not assessed as independent trials. For trial programs with multiple eligible publications, the RoB 2 assessment was applied to the PFS result used for the principal trial-level efficacy interpretation in this review rather than assigning separate risk-of-bias ratings to each publication. The resulting domain-level and overall RoB 2 judgments for PFS across the six included randomized trial programs are summarized in Table 1.

**Table 1:** Cochrane RoB 2 assessment of PFS NOTE: RoB 2 judgments refer specifically to the progression-free survival (PFS) result and the effect of assignment to intervention. CABOSUN was judged to have some concerns in Domain 5 (selection of the reported result) because the blinded independent radiology review used for the updated PFS analysis was initiated retrospectively after the original investigator-assessed primary PFS endpoint had been met; risk of bias in outcome measurement itself was judged low. JAVELIN Renal 101 remained at low risk because the protocol amendment occurred while treatment data were still masked. In ETER100, four of 531 randomized participants were excluded from the principal full analysis set, but prespecified intention-to-treat sensitivity analyses including all randomized participants produced results consistent with the primary analysis; therefore, this did not materially alter the risk-of-bias judgment.

Trial	Randomization Process	Deviations from Intended Interventions	Missing Outcome Data	Measurement of Outcome	Selection of Reported Result	Overall Judgment
CABOSUN	Low	Low	Low	Low	Some concerns	Some concerns
CheckMate 9ER	Low	Low	Low	Low	Low	Low
JAVELIN Renal 101	Low	Low	Low	Low	Low	Low
KEYNOTE-426	Low	Low	Low	Low	Low	Low
CLEAR	Low	Low	Low	Low	Low	Low
ETER100	Low	Low	Low	Low	Low	Low

The five RoB 2 domains evaluated were bias arising from the randomization process, bias due to deviations from intended interventions, bias due to missing outcome data, bias in measurement of the outcome, and bias in selection of the reported result. Domain-level and overall judgments were categorized as low risk, some concerns, or high risk of bias.

Five of the six trial programs were judged to have a low overall risk of bias for the PFS result: CheckMate 9ER, JAVELIN Renal 101, KEYNOTE-426, CLEAR, and ETER100. CABOSUN was judged to have some concerns because the blinded independent radiology committee PFS assessment reported in the updated analysis was conducted retrospectively, whereas the original primary PFS endpoint was investigator-assessed. In ETER100, the principal full analysis set excluded four of 531 randomized participants; however, this represented less than 1% of the randomized population, and prespecified sensitivity analyses using all randomized participants produced findings consistent with the primary analysis. No trial was judged to have a high overall risk of bias.

Results

Study Selection and Characteristics

Ten publications representing six unique clinical trials met the eligibility criteria and were included in the qualitative synthesis. The included publications evaluated sunitinib compared with contemporary TKI-based or TKI-containing first-line regimens in patients with untreated advanced or metastatic clear-cell RCC. Because CABOSUN evaluated a TKI monotherapy comparison whereas the remaining trial programs primarily evaluated TKI-containing combination regimens against sunitinib monotherapy, these two treatment strategies were considered separately in the synthesis. Table 2 summarizes the included publications, underlying trials, populations, treatment comparisons, and key outcomes. Safety outcomes were summarized separately by trial, including high-grade adverse events, treatment discontinuation, treatment-related mortality, dose modifications or interruptions, and immune-related adverse events when reported (Appendix 3).

**Table 2:** Trial-level summary of the six unique randomized clinical trials included in the review

Trial	Population	Treatment Comparison	Included Analyses	Main Finding
CABOSUN	Previously untreated advanced clear-cell RCC; IMDC intermediate/poor risk	Cabozantinib vs. sunitinib	Primary analysis; updated analysis; subgroup analysis	Cabozantinib improved PFS and ORR; OS difference was not statistically definitive.
CheckMate 9ER	Previously untreated advanced clear-cell RCC	Nivolumab + cabozantinib vs. sunitinib	Primary full-text analysis	Nivolumab plus cabozantinib improved PFS, OS, and ORR versus sunitinib in the included primary full- text analysis.
JAVELIN Renal 101	Previously untreated advanced RCC with a clear-cell component	Avelumab + axitinib vs. sunitinib	Primary analysis; final long-term analysis	PFS and ORR favored the combination; final OS difference was not statistically significant.
KEYNOTE-426	Previously untreated advanced clear- cell RCC	Pembrolizumab + axitinib vs. sunitinib	Primary global full-text analysis; East Asian subgroup analysis	Global trial demonstrated improved PFS, OS, and ORR; East Asian findings were supportive subgroup evidence.
CLEAR	Treatment-naïve advanced RCC, predominantly clear-cell histology	Lenvatinib + pembrolizumab vs. sunitinib	Final prespecified OS analysis	Combination demonstrated substantial PFS and ORR benefit and favored OS.
ETER100	Previously untreated advanced clear- cell RCC	Benmelstobart + anlotinib vs. sunitinib	Prespecified interim analysis	Combination improved PFS and ORR; OS remained immature.

Efficacy Outcomes

TKI monotherapy comparison: The CABOSUN trial compared cabozantinib with sunitinib in 157 previously untreated patients with intermediate- or poor-risk advanced clear-cell RCC [8]. The primary analysis demonstrated improved PFS and ORR with cabozantinib [8], and the subsequent independent-review analysis confirmed the PFS benefit, with median PFS of 8.6 versus 5.3 months [Hazard ratio (HR) 0.48; 95% confidence interval (CI), 0.31-0.74] [9]. Median OS numerically favored cabozantinib but was not statistically definitive [9]. A subsequent subgroup analysis generally demonstrated consistent PFS and ORR benefit across baseline characteristics, including IMDC risk group, bone metastases, age, and tumor burden [10].

TKI-containing combination regimens vs. sunitinib monotherapy: Choueiri et al. [11] evaluated nivolumab plus cabozantinib compared with sunitinib in the phase 3 CheckMate 9ER trial. The study included 651 patients with previously untreated advanced RCC with a clear-cell component, randomized to nivolumab plus cabozantinib (n=323) or sunitinib (n=328). Median PFS was significantly longer with nivolumab plus cabozantinib than with sunitinib (16.6 vs. 8.3 months; HR 0.51; 95% CI, 0.41-0.64; p<0.001). OS also favored nivolumab plus cabozantinib, with 12-month OS rates of 85.7% versus 75.6%, respectively (HR 0.60; 98.89% CI, 0.40-0.89; p=0.001). ORR was higher with nivolumab plus cabozantinib than with sunitinib (55.7% vs. 27.1%; p<0.001). Adverse events of any cause of grade 3 or higher occurred in 75.3% of patients receiving nivolumab plus cabozantinib and 70.6% receiving sunitinib. This trial provides strong evidence favoring nivolumab plus cabozantinib over sunitinib as a first-line TKI-containing regimen.

Motzer et al. [12] evaluated avelumab plus axitinib compared with sunitinib in patients with previously untreated advanced RCC with a clear-cell component. A total of 886 patients were randomized to receive avelumab plus axitinib (n=442) or sunitinib (n=444), including 560 patients with PD-L1-positive tumors. In the PD-L1-positive population, median PFS was longer with avelumab plus axitinib than with sunitinib (13.8 vs. 7.2 months; HR 0.61; 95% CI, 0.47-0.79; p<0.001). In the overall population, median PFS was also improved with avelumab plus axitinib (13.8 vs. 8.4 months; HR 0.69; 95% CI, 0.56-0.84). ORRs were higher with avelumab plus axitinib in both the PD-L1-positive population (55.2% vs. 25.5%) and the overall population (51.4% vs. 25.7%). OS data were immature at the time of analysis but numerically favored the combination. Adverse events of grade 3 or higher during treatment occurred in 71.2% of patients receiving avelumab plus axitinib and 71.5% receiving sunitinib. These results support avelumab plus axitinib as an effective first-line option for improving disease control compared with sunitinib.

Choueiri et al. [13] reported the final analysis of JAVELIN Renal 101, providing long-term follow-up of avelumab plus axitinib compared with sunitinib. With a minimum follow-up of 68 months, median OS in the overall population was 44.8 months with avelumab plus axitinib compared with 38.9 months with sunitinib, although this did not reach statistical significance (HR 0.88; 95% CI, 0.749-1.039; p=0.0669). PFS benefit was maintained, with a 5-year PFS rate of 12.0% with avelumab plus axitinib compared with 4.4% with sunitinib. ORR also remained higher with avelumab plus axitinib than with sunitinib (59.7% vs. 32.0%). Grade 3 or higher treatment-related AEs occurred in 66.8% of patients receiving avelumab plus axitinib and 61.5% receiving sunitinib. The final analysis supports the durability of avelumab plus axitinib benefit, while also showing that the OS advantage over sunitinib was not statistically definitive.

Rini et al. [14] evaluated pembrolizumab plus axitinib compared with sunitinib in patients with previously untreated advanced clear-cell RCC. A total of 861 patients were randomized to receive pembrolizumab plus axitinib (n=432) or sunitinib (n=429). At 12 months, OS was higher with pembrolizumab plus axitinib than with sunitinib (89.9% vs. 78.3%; HR 0.53; 95% CI, 0.38-0.74; p<0.0001). Median PFS was also longer with pembrolizumab plus axitinib (15.1 vs. 11.1 months; HR 0.69; 95% CI, 0.57-0.84; p<0.001). ORR was higher with the combination than with sunitinib (59.3% vs. 35.7%; p<0.001). Grade 3 or higher adverse events of any cause occurred in 75.8% of patients receiving pembrolizumab plus axitinib and 70.6% receiving sunitinib.

Motzer et al. [15] reported the final prespecified overall survival analysis of the phase III CLEAR trial evaluating lenvatinib plus pembrolizumab compared with sunitinib as first-line therapy in treatment-naïve advanced RCC. In the relevant treatment arms, 355 patients received lenvatinib plus pembrolizumab and 357 received sunitinib. With a median OS follow-up of approximately four years, the final OS analysis favored lenvatinib plus pembrolizumab (HR 0.79; 95% CI, 0.63-0.99), with 36-month OS rates of 66.4% versus 60.2%, respectively. The final prespecified OS analysis was descriptive and noninferential, with nominal P-values reported in the source publication. Median PFS was 23.9 months with lenvatinib plus pembrolizumab compared with 9.2 months with sunitinib (HR 0.47; 95% CI, 0.38-0.57), and ORR was 71.3% versus 36.7%. Grade ≥3 treatment-emergent adverse events occurred in 84.9% and 74.7% of patients, respectively. These mature findings support lenvatinib plus pembrolizumab as an effective first-line TKI-containing combination while highlighting the substantial toxicity burden observed during treatment.

Zhou et al. [16] evaluated benmelstobart plus anlotinib compared with sunitinib in 531 patients with untreated advanced clear-cell RCC. Patients were randomized to receive benmelstobart plus anlotinib (n=266) or sunitinib (n=265). Median PFS was significantly longer with benmelstobart plus anlotinib than with sunitinib (19.0 vs. 9.8 months; HR 0.53; 95% CI, 0.42-0.67; p<0.0001). ORR was also higher with the combination (72% vs. 25%; p<0.0001). OS data were not mature, although early results favored benmelstobart plus anlotinib. Grade 3 or worse treatment-related adverse events occurred in 67% of patients receiving benmelstobart plus anlotinib and 66% receiving sunitinib. These findings suggest that benmelstobart plus anlotinib offers improved disease control compared with sunitinib, although OS data remain immature.

Chung et al. [17] reported an East Asian subgroup analysis of the phase 3 KEYNOTE-426 trial comparing pembrolizumab plus axitinib with sunitinib. The analysis included 130 patients with untreated clear-cell metastatic RCC from Japan, South Korea, and Taiwan. Median PFS was longer with pembrolizumab plus axitinib than with sunitinib (20.6 vs. 10.0 months; HR 0.59; 95% CI, 0.38-0.92). OS favored pembrolizumab plus axitinib, although the estimate was limited by subgroup size (47.2 vs. 44.2 months; HR 0.85; 95% CI, 0.50-1.44). ORR was higher with pembrolizumab plus axitinib than with sunitinib (64.5% vs. 44.1%). Grade 3-4 treatment-related adverse events occurred in 69.4% of patients receiving pembrolizumab plus axitinib and 74.6% receiving sunitinib. These findings provide supportive regional subgroup evidence but should not be interpreted as an independent randomized comparison or as confirmatory evidence separate from the global KEYNOTE-426 trial.

Safety and Generalizability

Across the included publications, contemporary TKI-based and TKI-containing first-line regimens consistently demonstrated improved PFS compared with sunitinib, and ORRs were also higher in nearly all within-trial comparisons. OS benefit was more variable across the included trials. However, absolute outcomes should not be directly compared between separate trials because of differences in IMDC risk distribution, eligibility criteria, follow-up duration, endpoint assessment, and subsequent therapies. Accordingly, interpretation in this review emphasizes within-trial treatment effects, particularly hazard ratios and corresponding confidence intervals, rather than numerical comparisons across unrelated trial populations. Reported high-grade adverse-event rates were common across experimental and sunitinib arms; however, these rates were not directly combined or compared across trials because the reported definitions differed. Specifically, individual studies reported grade 3-4 or grade ≥3 events using all-causality, treatment-emergent, or treatment-related definitions. These categories were preserved as reported by the original studies and were not treated as interchangeable.

Improved efficacy was accompanied by clinically important toxicity across the included regimens. High-grade adverse events were frequent, and treatment discontinuations, dose modifications, and treatment-related deaths were reported in several trials. ICI-TKI combinations also introduced immune-related adverse events, while common toxicities reported in individual studies included diarrhea, hypertension, and thyroid disorders. These safety considerations should therefore be weighed alongside efficacy when selecting first-line therapy.

Discussion

Sunitinib historically served as a standard first-line VEGF-targeted therapy for metastatic clear-cell RCC [18]. However, its role has declined because of limitations including toxicity and acquired resistance, alongside the emergence of newer treatment strategies [19-22]. The included evidence therefore reflects a broader transition from sunitinib monotherapy toward alternative TKI monotherapy in selected patients and, more prominently, ICI-TKI combination regimens.

TKI Monotherapy Evidence

CABOSUN supports cabozantinib as an alternative to sunitinib in patients with intermediate- or poor-risk disease. The primary and updated analyses demonstrated improved PFS and ORR with cabozantinib, while the OS difference was not statistically definitive. Subgroup analyses generally supported this benefit across baseline characteristics; however, the trial’s small sample size, exclusion of favorable-risk patients, and exploratory nature of the subgroup analyses limit generalizability [8-10].

TKI-Containing Combination Regimens vs. Sunitinib Monotherapy

Pembrolizumab plus axitinib demonstrated improved efficacy compared with sunitinib in the primary KEYNOTE-426 analysis, with benefits in OS, PFS, and ORR. However, the included publication had a median follow-up of 12.8 months, limiting assessment of mature long-term efficacy and safety within the evidence set captured by this review [14].

CheckMate 9ER demonstrated improved PFS, OS, and ORR with nivolumab plus cabozantinib compared with sunitinib. However, the included publication represents the primary analysis, limiting assessment of long-term durability within the evidence set captured by this review [11].

The CLEAR trial further supports the transition toward TKI-containing immunotherapy combinations. Lenvatinib plus pembrolizumab demonstrated durable improvements in PFS and ORR compared with sunitinib, while the final prespecified OS analysis favored the combination. However, the substantial rate of high-grade adverse events highlights the importance of balancing efficacy with treatment-related toxicity when selecting first-line therapy [15].

JAVELIN Renal 101 demonstrated improved PFS and ORR with avelumab plus axitinib compared with sunitinib. OS data were immature in the primary analysis, while the included final long-term analysis did not demonstrate a statistically significant OS advantage over sunitinib. The frequency of immune-related adverse events further highlights the importance of toxicity management when considering this regimen [12,13].

ETER100 similarly demonstrated improved PFS and ORR with benmelstobart plus anlotinib compared with sunitinib. However, OS data remain immature, and treatment-related adverse events, discontinuations, and treatment-related deaths emphasize the need for longer-term efficacy and safety follow-up [16].

Comparative Interpretation of First-Line Regimens

Importantly, the newer regimens evaluated in this review should not be considered clinically equivalent or supported by identical levels of evidence. Pembrolizumab plus axitinib, nivolumab plus cabozantinib, and lenvatinib plus pembrolizumab represent established first-line treatment options; however, the maturity of OS evidence available within the included publication set differs among them. CLEAR provides mature, final prespecified OS evidence favoring lenvatinib plus pembrolizumab over sunitinib. In contrast, the eligible CheckMate 9ER and KEYNOTE-426 publications captured by this review represent primary analyses and therefore provide less mature long-term OS evidence within the present synthesis. Avelumab plus axitinib demonstrated durable PFS and ORR benefit, but the final JAVELIN Renal 101 analysis did not demonstrate a statistically significant OS advantage over sunitinib. Benmelstobart plus anlotinib demonstrated promising PFS and ORR results in ETER100, but OS remained immature and broader international applicability remains uncertain. These differences in evidence maturity and clinical status should be considered when interpreting the included regimens.

Overall, the included trials demonstrate that contemporary first-line regimens generally provide improved progression-free survival and tumor response compared with sunitinib monotherapy. Because these regimens were evaluated in separate randomized trials with differing patient populations, eligibility criteria, follow-up durations, and endpoint assessments, their absolute PFS and ORR values should not be used to rank one regimen against another. No comparative hierarchy among the regimens can be established from this review in the absence of head-to-head trials or an appropriately conducted network meta-analysis. However, these findings should not be interpreted as demonstrating that the alternative TKI component itself is superior to sunitinib. CABOSUN directly compared cabozantinib monotherapy with sunitinib monotherapy, whereas most of the other included trials compared a TKI-containing immune checkpoint inhibitor combination with sunitinib alone. Therefore, the improved outcomes observed in the combination trials reflect the efficacy of the complete treatment regimen and cannot isolate the independent contribution of the alternative TKI from that of the immune checkpoint inhibitor.

Overall survival benefit was more variable and appeared to be influenced by follow-up duration, subsequent therapies, and trial population. Tolerability and patient-specific factors such as age, comorbidities, performance status, IMDC risk group, histology, and biomarkers also influence treatment selection and real-world effectiveness.

Limitations of the Included Evidence

Study limitations, including small sample sizes, short follow-up periods, subgroup analyses, and selective trial populations, warrant caution when generalizing these findings beyond the populations studied.

Limitations of This Review

Additionally, because study screening, eligibility assessment, and data extraction were performed by a single reviewer, there is a potential risk of selection bias and extraction error. Although database coverage was expanded to include PubMed and Embase, additional databases and clinical-trial registries were not systematically searched; therefore, relevant publications may have been missed. Despite these limitations, the evidence supports the transition from sunitinib monotherapy toward contemporary TKI-based and TKI-containing first-line regimens, while recognizing that the combination trials evaluate the efficacy of the overall regimen rather than the isolated contribution of the TKI component.

Future directions

Future research should focus on longer-term survival and quality-of-life outcomes, treatment selection and sequencing after first-line ICI-TKI therapy, and validation of biomarkers that may guide individualized treatment. Additional studies in underrepresented populations are also needed to improve the generalizability of treatment-selection strategies across different IMDC risk groups, performance-status categories, and patient characteristics.

## Conclusions

In conclusion, sunitinib has been displaced as the preferred first-line standard for most patients with metastatic clear-cell RCC as newer TKI-based and TKI-containing regimens have demonstrated improved clinical outcomes; however, it has not become obsolete. Across the included publications, cabozantinib monotherapy and TKI-containing combination regimens generally improved progression-free survival and objective response rates compared with sunitinib, while overall survival benefit varied across trials. Because most included comparisons evaluated TKI-containing combination therapy against sunitinib monotherapy, these findings support the relative efficacy of the complete modern treatment regimens rather than establishing intrinsic superiority of the alternative TKI component over sunitinib. Toxicities were common across both experimental and sunitinib arms and varied according to the safety definitions and treatment regimens evaluated. The strength and maturity of evidence varied among the included regimens, reinforcing the need for individualized first-line treatment selection. Among the regimens reviewed, pembrolizumab plus axitinib, nivolumab plus cabozantinib, and lenvatinib plus pembrolizumab are established first-line options; avelumab plus axitinib did not demonstrate a statistically significant OS advantage at final analysis, while benmelstobart plus anlotinib remains supported by immature OS data and regionally limited evidence. Sunitinib nevertheless remains an evidence-based option in selected clinical circumstances, including contraindications to immune checkpoint inhibition, selected patients with favorable-risk disease, and limited access to combination therapy. Treatment selection should therefore remain individualized according to IMDC risk category, performance status, comorbidities, toxicity profile, treatment access, and other patient-specific considerations.

## Appendices

Appendix 1 

**Table 3:** Trial design and study population NOTE: For CLEAR, the sample size shown refers to the lenvatinib plus pembrolizumab versus sunitinib comparison relevant to this review. Multiple publications arising from the same randomized trial are grouped under the corresponding trial and are not treated as independent patient populations. The KEYNOTE-426 East Asian publication represents a subgroup analysis of the global trial.

Trial	Included Analyses	Study Design	Sample Size	Study Population / Histology	Risk / Performance Status	Treatment Comparison
CABOSUN	Primary analysis; updated analysis; post hoc subgroup analysis	Randomized controlled trial	157	Previously untreated advanced or metastatic RCC with a clear-cell component; measurable disease	IMDC intermediate or poor risk; ECOG PS 0–2	Cabozantinib (n=79) vs. sunitinib (n=78)
CheckMate 9ER	Primary full-text analysis	Randomized controlled trial	651	Previously untreated advanced RCC with a clear-cell component	IMDC favorable, intermediate, and poor risk; KPS 70–100	Nivolumab + cabozantinib (n=323) vs. sunitinib (n=328)
JAVELIN Renal 101	Primary analysis; final long-term analysis	Randomized controlled trial	886	Previously untreated advanced RCC with a clear-cell component	ECOG PS 0–1; prognostic risk groups represented	Avelumab + axitinib (n=442) vs. sunitinib (n=444)
KEYNOTE-426	Primary global analysis; East Asian subgroup analysis	Randomized controlled trial	861	Previously untreated advanced clear-cell RCC	IMDC favorable, intermediate, and poor risk; KPS ≥70	Pembrolizumab + axitinib (n=432) vs. sunitinib (n=429)
CLEAR	Final prespecified OS analysis	Randomized controlled trial	712*	Treatment-naïve advanced RCC, predominantly clear- cell histology	IMDC favorable, intermediate, and poor risk groups included	Lenvatinib + pembrolizumab (n=355) vs. sunitinib (n=357)
ETER100	Prespecified interim analysis	Randomized controlled trial	531 randomized; 527 in full analysis set	Patients aged 18–80 years with previously untreated advanced clear-cell RCC; enrolled at 37 sites in China	IMDC favorable, intermediate, and poor risk; ECOG PS 0–1	Benmelstobart + anlotinib (n=266 randomized) vs. sunitinib (n=265 randomized)

Appendix 2 

**Table 4:** Trial-level efficacy outcomes NOTE: Outcomes are presented for the experimental regimen versus sunitinib. When multiple eligible publications arose from the same trial, the most mature eligible analysis captured by this review was prioritized for long-term efficacy interpretation. Earlier analyses are included when needed to report outcomes not provided in the later publication. CheckMate 9ER and KEYNOTE-426 values reflect the eligible primary global full-text analyses included in this review. Detailed subgroup findings are not treated as independent trial evidence.

Trial	Analysis/Follow-up	Progression-Free Survival (PFS)	Overall Survival (OS)	Objective Response Rate (ORR)	Key Interpretation
CABOSUN	Updated analysis; median follow-up 34.5 months	8.6 vs. 5.3 months; HR 0.48 (95% CI, 0.31–0.74); p=0.0008	26.6 vs. 21.2 months; HR 0.80 (95% CI, 0.53-1.21)	20% vs. 9%	Cabozantinib improved PFS and ORR; OS difference was not statistically definitive.
CheckMate 9ER	Primary full-text analysis; median OS follow-up 18.1 months	16.6 vs. 8.3 months; HR 0.51 (95% CI, 0.41–0.64); p<0.001	12-month OS: 85.7% vs. 75.6%; HR 0.60 (98.89% CI, 0.40–0.89); p=0.001	55.7% vs. 27.1%; p<0.001	Nivolumab + cabozantinib improved PFS, OS, and ORR in the included primary analysis.
JAVELIN Renal 101	Primary and final analyses; minimum final follow-up 68 months	Primary analysis: 13.8 vs. 8.4 months in the overall population; HR 0.69 (95% CI, 0.56–0.84). Final 5-year PFS: 12.0% vs. 4.4%.	Final median OS: 44.8 vs. 38.9 months; HR 0.88 (95% CI, 0.749–1.039); p=0.0669	Final ORR: 59.7% vs. 32.0%	PFS and ORR benefits were maintained with long-term follow-up; final OS difference was not statistically significant.
KEYNOTE-426	Primary global full-text analysis; median follow-up 12.8 months	15.1 vs. 11.1 months; HR 0.69 (95% CI, 0.57–0.84); p<0.001	12-month OS: 89.9% vs. 78.3%; HR 0.53 (95% CI, 0.38–0.74); p<0.0001	59.3% vs. 35.7%; p<0.001	The included primary global analysis favored pembrolizumab + axitinib across PFS, OS, and ORR.
CLEAR	Final prespecified OS analysis; median OS follow-up approximately 4 years	23.9 vs. 9.2 months; HR 0.47 (95% CI, 0.38–0.57)	HR 0.79 (95% CI, 0.63–0.99); 36- month OS 66.4% vs. 60.2%	71.3% vs. 36.7%	Lenvatinib + pembrolizumab demonstrated substantial PFS and ORR benefit, with final OS favoring the combination.
ETER100	Prespecified interim analysis; median follow-up 22.8 months	19.0 vs. 9.8 months; HR 0.53 (95% CI, 0.42–0.67); p<0.0001	Immature at the reported analysis	72% vs. 25%; p<0.0001	Benmelstobart + anlotinib improved PFS and ORR; mature OS data were unavailable.

Appendix 3 

**Table 5:** Trial-level summary of reported safety outcomes NOTE: Safety outcomes are reported using the terminology of the included source publications because adverse-event definitions differed among trials. All-causality, treatment-emergent, and treatment-related adverse events were therefore not treated as interchangeable. When multiple included publications arose from the same trial, the analysis providing the relevant safety outcome was used. NR indicates that the outcome was not reported in the included source used for that safety assessment. AE, adverse event; TEAE, treatment-emergent adverse event; TRAE, treatment-related adverse event

Trial	High-grade adverse events	Discontinuation due to adverse events	Treatment-related deaths	Dose modifications/ interruptions	Immune-related or other notable safety findings	Key limitations / generalizability
CABOSUN	Grade 3–4 all-causality AEs: 68% cabozantinib vs. 65% sunitinib	Discontinuation due to an AE: 21% vs. 22%	Two grade 5 AEs considered related to cabozantinib; four related to sunitinib	Dose reductions: 46% vs. 35%	Not applicable; neither treatment contained an immune checkpoint inhibitor	Small trial (n=157); restricted to IMDC intermediate- and poor-risk patients, limiting generalizability to favorable-risk disease
CheckMate 9ER	Grade ≥3 AEs of any cause: 75.3% nivolumab + cabozantinib vs. 70.6% sunitinib	Discontinuation due to AEs: 19.7% with nivolumab plus cabozantinib vs. 16.9% with sunitinib.	NR in the included source record	NR in the included source record	NR in the included source record	Included evidence is based on the primary full-text analysis with 18.1 months median OS follow-up; trial eligibility favored patients with adequate performance status
JAVELIN Renal 101	Final analysis: grade ≥3 treatment-related AEs: 66.8% avelumab + axitinib vs. 61.5% sunitinib	Primary analysis: AEs during treatment led to discontinuation of both avelumab and axitinib in 7.6% vs. discontinuation of sunitinib in 13.4%	Death due to toxicity of trial treatment: 0.7% vs. 0.2%	At least one axitinib dose reduction: 42.2%; sunitinib dose reduction: 42.6%	Immune-related AEs occurred in 38.2%; grade ≥3 in 9.0%; immune-related thyroid disorders in 24.7%	ECOG PS 0–1 population may limit applicability to frailer patients; final OS difference was not statistically significant
KEYNOTE-426	Grade ≥3 treatment-related AEs: 62.9% pembrolizumab + axitinib vs. 58.1% sunitinib. Grade ≥3 AEs of any cause: 75.8% vs. 70.6%	AEs of any cause led to discontinuation of either combination drug in 30.5%, both combination drugs in 10.7%, and sunitinib in 13.9%	Treatment-related AE deaths: 0.9% vs. 1.6%	Interruption of either combination drug: 69.9%; sunitinib: 49.9%. Axitinib dose reduction: 20.3%; sunitinib: 30.1%	Prespecified AEs of interest occurred in 51.3% vs. 36.2%; assessed regardless of investigator-attributed causality	Included global analysis had 12.8 months median follow-up; East Asian publication is a subgroup analysis and should not be interpreted as independent global evidence
CLEAR	Grade ≥3 treatment-emergent AEs: 84.9% lenvatinib + pembrolizumab vs. 74.7% sunitinib	NR in the included final OS publication	Fatal treatment-related AEs: 1.1% vs. 0.3%	NR in the included final OS publication	Diarrhea was the most common TEAE and hypertension the most common grade ≥3 TEAE; detailed immune-mediated event rates were not reported in the included final OS publication	Predominantly clear-cell trial population; substantial treatment-emergent toxicity burden; several detailed safety outcomes were not reported in the included final OS publication
ETER100	Grade ≥3 treatment-related AEs: 67% benmelstobart + anlotinib vs. 66% sunitinib; serious treatment-related AEs: 24% vs. 16%	Treatment-related AEs led to discontinuation of benmelstobart in 7%, anlotinib in 8%, and sunitinib in 4%	Three deaths (1%) with benmelstobart + anlotinib were considered treatment related; none reported for sunitinib	Dose reduction due to TRAEs: anlotinib 34% vs. sunitinib 47%. Treatment interruption: benmelstobart 39%, anlotinib 45%, sunitinib 51%	Immune-related AEs occurred in 39%; grade ≥3 in 11%; 4% required systemic high-dose corticosteroids	Conducted exclusively in a Chinese population, limiting geographic generalizability; OS data remained immature
